# Random forest model used to predict the medical out-of-pocket costs of hypertensive patients

**DOI:** 10.3389/fpubh.2024.1382354

**Published:** 2024-07-17

**Authors:** Narimasa Kumagai, Mihajlo Jakovljević

**Affiliations:** ^1^Faculty of Economics, Seinan Gakuin University, Fukuoka, Japan; ^2^UNESCO-TWAS, Section of Social and Economic Sciences, Trieste, Italy; ^3^Shaanxi University of Technology, Hanzhong, China; ^4^Department of Global Health Economics and Policy, University of Kragujevac, Kragujevac, Serbia

**Keywords:** activities of daily living (ADL), exercise, hypertension, Japan, out-of-pocket (OOP) costs, prediction, random forest

## Abstract

**Background:**

Precise prediction of out-of-pocket (OOP) costs to improve health policy design is important for governments of countries with national health insurance. Controlling the medical expenses for hypertension, one of the leading causes of stroke and ischemic heart disease, is an important issue for the Japanese government. This study aims to explore the importance of OOP costs for outpatients with hypertension.

**Methods:**

To obtain a precise prediction of the highest quartile group of OOP costs of hypertensive outpatients, we used nationwide longitudinal data, and estimated a random forest (RF) model focusing on complications with other lifestyle-related diseases and the nonlinearities of the data.

**Results:**

The results of the RF models showed that the prediction accuracy of OOP costs for hypertensive patients without activities of daily living (ADL) difficulties was slightly better than that for all hypertensive patients who continued physician visits during the past two consecutive years. Important variables of the highest quartile of OOP costs were age, diabetes or lipidemia, lack of habitual exercise, and moderate or vigorous regular exercise.

**Conclusion:**

As preventing complications of diabetes or lipidemia is important for reducing OOP costs in outpatients with hypertension, regular exercise of moderate or vigorous intensity is recommended for hypertensive patients that do not have ADL difficulty. For hypertensive patients with ADL difficulty, habitual exercise is not recommended.

## 1 Introduction

Given the poverty caused by rising health care costs for chronically ill patients, accurate prediction of out-of-pocket (OOP) costs is important to prevent catastrophic health care expenditures for those patients because OOP costs and prepaid private health financing are also expected to grow, although less than growth in government spending (1). On the one hand, research by Hwang et al. (2) showed a positive, nearly linear relationship between OOP costs and the number of chronic diseases. On the other hand, Zhang et al. (3) used a data-driven ensemble learning procedure and found that the top-ranking factors that best predicted OOP costs were insurance type, age, asthma, family size, race, and number of physician visits.

Although three or more activities of daily living (ADL) dependencies are associated with the need for long-term care, the onset of ADL difficulties in chronically ill persons may differ from that of healthy older adults (4). Therefore, both ADL difficulties and major chronic diseases should be considered when analyzing physician visits and predicting OOP costs.

Hypertension is the leading cause of stroke and ischemic heart disease, leading to premature death in Japan and worldwide (5–7). Individuals with well-controlled hypertension who are undergoing treatment have a lower risk of developing cardiovascular or cerebrovascular diseases (8, 9). In Japan, where most hypertensive patients tend to adhere to their family physicians’ treatment plans, regular physician visits every 30 days are effective in stabilizing blood pressure in hypertensive patients (10).

The prevalence of hypertension control among hypertensive patients taking antihypertensive medications increased from 1980 to 2016 in Japan (11). Furthermore, 72% (31 million) of the hypertensive patients were poorly controlled in 2017. Poorly controlled hypertension during treatment is expensive. A study by Japanese researchers showed that grade 3 untreated hypertension is likely to be extremely costly (12). Hence, controlling blood pressure in patients with hypertension through physician visits and medical expenses is an important issue for the Japanese government. The estimates of the average treatment effects on treated patients suggest that three consecutive years of physician visits negatively impacted the poor subjective health of hypertensive patients (10). Therefore, this study focuses on the patients who had physician visits during the past two consecutive years.

This study estimates the predicted OOP costs of outpatients in Japan with hypertension who are middle-aged and older people. We used data from a longitudinal survey conducted by the Ministry of Health (2005–2020) and estimated a random forest (RF) model focusing on complications with other lifestyle-related diseases and nonlinearities. The Stata command “rforest” in Stata version 18 (StataCorp) was used to estimate RF models.

Can the governments of countries with national health insurance precisely predict OOP costs and improve health policy design? The challenge of fiscal sustainability of healthcare financing due to increased OOP expenditures is particularly alarming in the Global South’s low- and middle-income countries (LMICs) worldwide (13). This is because of huge socioeconomic inequalities as shown by GINI indices and consecutive poor affordability of medical care (14), particularly in the vast rural and remote peripheries of these nations (15). This imbalance in health expenditure dynamics was created, to a large extent, through the decade-long evolution of the morbidity landscape, much earlier in industrialized wealthy societies of the Global North (16). Namely, traditional, mostly curable infectious diseases of the short clinical course (17) were gradually replaced by incurable, life-time chronic noncommunicable diseases (NCDs) such as hypertension, diabetes and all consequences of atherosclerosis (18). Such disorders remain expensive and difficult to treat, imposing a far higher burden to the health system. Risk sharing occurring in early modern health systems of the late XIX and early XX century have resolved this burden only to a limited extent (19). Technological innovation (20) and significant life expectancy extension worldwide associated with population aging have only worsened the situation (21). Therefore, health econometric exploration of the underlying causes in large, aged societies of Asia (22) remain capable of revealing hidden, underlying patterns of spending (23). They may also provide glimpses of possible policy strategies targeted at both prevention and more effective technical and allocative efficacy of provision and delivery of medical care (24). We believe that our study makes a significant practical contribution to the prediction of OOP costs and, therefore, the improvement of national health insurance and health policy design.

The remainder of this paper is organized as follows: Section 2 provides an overview of the machine learning method, RF, and its split selection based on an ensemble learning algorithm. Section 3 presents the estimation results of the RF model. Section 4 discusses the variable importance of OOP costs for outpatients with hypertension. Section 5 concludes the paper.

## 2 Methods

### 2.1 Random forest model

RF has the advantage of treating missing values because splits at any node can occur even if some independent variables are missing. Very few restrictions are imposed on the choice of explanatory variables because no functional form is assumed (25). Using an RF model, we can predict the dichotomous variable and obtain the predictions with no significant bias (26). The RF model does not estimate the coefficients of the explanatory variables in the same way as econometric models and easily adapts to the nonlinearities found in the data; therefore, it tends to predict better than linear regression (27). Researchers can use several explanatory variables to eliminate the effects of overtraining. The RF estimate was computed with a minimum leaf size of 1 and 800, averaged over the results of 100 trees with no maximum depth (these are the defaults for rforest). The full model has 45 features.

We split a nationally representative sample of the older adults in Japan which is explained below into two subsets: 50% of the data are used for training, and 50% of the data are used for validation. When an RF model randomly selects explanatory variables from among all the explanatory variables, the sample of the regression tree is split. RF uses entropy for split selection in classification cases. At each internal node of the decision tree, entropy (E) is given by Equation (1).


(1) $$E=-\sum_{i=1}^{c}p_{i}\times \log(p_{i})$$

where *c* is the number of unique classes, and *pi* is the prior probability for each class (27).

Each tree is constructed using different bootstrap samples. Each bootstrap sample randomly leaves out approximately one-third of the observations. These are referred to as the out-of-bag (OOB) samples. For classification problems, the OOB error used for validation represents the classification error. Thus, we can use an OOB error tested against training data subsets that are not included in subtree construction. The RF model analysis aims to explore the extent to which explanatory variables affect the dependent variable, and the effects are assessed using variable importance. Ranking the importance of the factors is challenging in data analysis. In supervised learning there are problems with multiple factors. Ranking the importance of factors involves assessing the impact of each factor on the response variable and creating quantitative measures for comparison (3).

Generally, variable importance, which measures the magnitude of the forecast error when explanatory variables are randomly selected, is used. The larger the prediction error, the greater the importance of the variables that can be evaluated. Variable importance is calculated by adding the improvement in the objective function given in the splitting criterion over all internal nodes of a tree and across all trees in the forest. The variable importance score is normalized by dividing all scores by the maximum score (27).

### 2.2 Data

We used longitudinal data over 16 consecutive years (2005–2020), obtained from the Longitudinal Survey of Middle and Older Persons (LSMOP) of the Japanese Ministry of Health, Labor, and Welfare (MHLW). Data were collected using a combination of interviews and self-administered questionnaires. The LSMOP collects information on family situation, health status, and employment status. In the second-wave of the survey, the LSMOP asked about the educational attainment of the respondents and their spouses.

The samples for the LSMOP were randomly selected using a two-stage sampling procedure. First, 2,515 districts in 2005 were randomly selected from the 5,280 districts covered by the “Comprehensive Survey of Living Conditions” conducted by the MHLW in 2004 (another nationwide survey). Second, 40,877 respondents aged 50–59 years, as of October 30, 2005, were randomly selected from each district.

The proportion of the total sum of 16 consecutive respondents to the subjects who responded to the second wave of the survey in 2006 was approximately 53% (10). A total of 34,240 individuals responded to the first wave of the survey (response rate: 83.8%), whereas 32,285 participants returned the questionnaires for the second wave (response rate: 92.2%). Almost half of the respondents in the 2006 survey dropped out of the study.

The sample in this study consisted of hypertensive patients who had continued physician visits during the past two consecutive years. Male patients and those with diabetes, lower educational attainment, no habitual exercise, or smoking habits tended not to have physician visits during the past two consecutive years (10).

Based on a doctor’s diagnosis, hypertension was defined as a systolic blood pressure (BP) ≥ 140 mmHg, a diastolic BP ≥ 90 mmHg, or use of antihypertensive medication. The hypertension control rate was defined as the proportion of patients with systolic BP < 140 mmHg and diastolic BP < 90 mmHg among the hypertensive patients taking antihypertensive medication (11). The proportion of patients with cancer, diabetes, heart disease, lipidemia, and strokes among the older adults with hypertension is higher than among the older adults without hypertension (10).

## 3 Results

**Figure 1:**
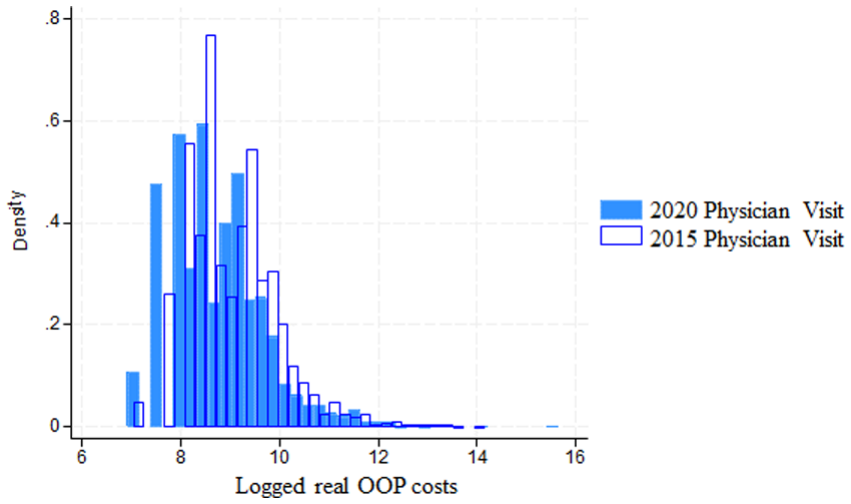
Logged real OOP costs for hypertensive patients (2020 vs 2015).

Figure 1 shows the logged real OOP costs in 2020 for hypertensive patients who had physician visits during 2019–20 (blue). It further shows hypertensive patients who had physician visits during 2014–15 (white). Two changes can be seen: first, the peak of the blue histogram is located more toward the left than the white histogram; and second, the tail of the right hem of the blue graph is getting longer as the proportion of patients aged 70–74 with a 20% co-payment rate is higher in 2020 than in 2015. This is associated with a shift to the left side of the histogram. Hypertensive patients in their 60s, whose co-payment rate was 30% more, contributed the right hem having a longer tail than the 70–74 age group in 2020. We pay attention to these points when interpreting the statistical data in Table 1.

**Table 1:** Sample characteristics of hypertensive patients who continued physician visits during the past two consecutive years.

	Highest quartile of OOP costs				All	
Variables	*N*	Mean or proportion	SD	*N*	Mean or proportion	SD
Dependent variable						
Logged real out-of-pocket (OOP) costs (2020 = 100)				57,710	9.093	0.999
Demographic variables						
Age	14,247	**63.73**	4.16	57,710	64.48	4.48
Dummy variable for aged 70–74	14,247	**0.089**	0.284	57,710	0.149	0.356
Gender (male = 1)	14,247	**0.542**	0.498	57,710	0.523	0.499
Married (reference)	14,247	**0.917**	0.275	57,710	0.945	0.227
Never married	14,247	**0.082**	0.275	57,710	0.055	0.227
Divorced or widowed	14,247	**0.000**	0.017	57,710	0.000	0.009
Dummy variable for living together with family members excluding spouse	14,246	**0.555**	0.497	57,693	0.539	0.498
Dummy variable for earned income during the past month	11,968	**0.578**	0.494	50,241	0.584	0.493
Educational attainment						
Junior high school	14,247	**0.172**	0.378	57,710	0.162	0.368
High school (reference)	14,247	**0.497**	0.500	57,710	0.514	0.500
Vocational school or junior college	14,247	0.145	0.352	57,710	0.144	0.351
University or graduate school	14,247	0.179	0.383	57,710	0.175	0.380
Sum of K6						
Serious mental health (12 < K6)	10,766	**0.085**	0.279	45,867	0.054	0.225
Moderate mental health (4 < K6 < 13)	10,766	**0.313**	0.464	45,867	0.244	0.429
Objective health status						
Dummy variable for having diabetes	14,059	**0.306**	0.461	56,997	0.196	0.397
Dummy variable for having heart diseases	14,004	**0.152**	0.359	56,790	0.092	0.289
Dummy variable for having lipidemia	13,946	**0.326**	0.469	56,712	0.292	0.454
Dummy variable for having stroke	13,884	**0.075**	0.263	56,274	0.043	0.202
Dummy variable for having cancer	13,903	**0.090**	0.286	56,533	0.042	0.200
Perceived health						
Dummy variable for felt worse symptoms of high blood pressure than its onset during the past year	14,247	**0.017**	0.129	57,710	0.009	0.093
Lifestyle						
Drinking habit	14,247	**0.249**	0.432	57,710	0.237	0.425
No habitual exercise	14,221	**0.346**	0.476	57,604	0.325	0.468
Moderate or vigorous regular exercise	14,247	**0.280**	0.449	57,710	0.307	0.461
Smoking habit	14,247	0.156	0.363	57,710	0.154	0.361
Having difficulties in activities of daily living (ADL)						
Dummy variable for walking	14,247	0.230	0.421	57,710	0.151	0.358
Dummy variable for getting in and out of bed	14,247	0.234	0.423	57,710	0.153	0.360
Dummy variable for standing up from a chair	14,247	0.234	0.424	57,710	0.153	0.360
Dummy variable for dressing	14,247	0.232	0.422	57,710	0.152	0.359
Dummy variable for washing face	14,247	0.234	0.424	57,710	0.153	0.360
Dummy variable for eating meals	14,247	0.235	0.424	57,710	0.153	0.360
Dummy variable for using the toilet	14,247	0.234	0.423	57,710	0.153	0.360
Dummy variable for bathing and showering	14,247	0.226	0.418	57,710	0.149	0.356
Dummy variable for stair climbing	14,247	0.225	0.417	57,710	0.148	0.356
Dummy variable for carrying of shopped items	14,247	0.213	0.410	57,710	0.144	0.351
Needing assistance for ADL						
Dummy variable for walking	14,247	0.009	0.096	57,710	0.004	0.064
Dummy variable for getting in and out of bed	14,247	0.005	0.073	57,710	0.002	0.046
Dummy variable for standing up from a chair	14,247	0.005	0.070	57,710	0.002	0.046
Dummy variable for dressing	14,247	0.008	0.087	57,710	0.003	0.056
Dummy variable for washing face	14,247	0.005	0.069	57,710	0.002	0.048
Dummy variable for eating meals	14,247	0.004	0.064	57,710	0.002	0.042
Dummy variable for using the toilet	14,247	0.005	0.073	57,710	0.002	0.047
Dummy variable for bathing and showering	14,247	0.013	0.113	57,710	0.006	0.074
Dummy variable for stair climbing	14,247	0.015	0.120	57,710	0.006	0.080
Dummy variable for carrying of shopped items	14,247	0.026	0.159	57,710	0.011	0.105

Non-respondents of OOP costs are excluded. Boldface indicates that the mean or the proportion of variables in the highest quartile group of copayments is statistically different from those of comparison group at the 1% level. Source: Longitudinal Survey of Middle-aged and Older Persons 2005–2020.

Table 1 shows the sample characteristics of the hypertensive patients who continued their physician visits during the past two consecutive years. The proportion of aged 70–74 in the highest quartile group of OOP costs was 0.089, which was significantly different from those of comparison group at the 1% level. The mean OOP cost in the highest quartile was approximately 38,900 Japanese yen per month (data not shown). By contrast, the proportion of patients with chronic diseases in the highest quartile group of OOP costs, such as diabetes, heart disease, stroke, and cancer, was much higher than that of the overall sample. Therefore, we conjectured that the number of chronic diseases contributes to higher OOP costs.

Approximately 18% of hypertensive patients who continued physician visits during the past two consecutive years did not respond to questions regarding their OOP costs at the time of the survey. Compared with the overall sample, a higher percentage of non-respondents were never married, less educated, had a serious mental health status, had no habitual exercise, and smoked (Supplementary Table S1). The number of variables to randomly investigate was the square root of the number of independent variables, and the number of iterations was 500.

To predict logged real OOP costs of hypertensive outpatients, the following 45 candidate covariates were included: age, gender, dummy variable for aged 70–74 as the proxy variable of lower copayment rate (20%), marital status, earned income during the past month, educational attainment, living together with family members excluding spouse, non-communicable diseases (coded as 5 dummy variables) such as having diabetes, mental health variables, perceived health, having difficulties in ADL or needing assistance for ADL (coded as 20 dummy variables), lifestyle variables such as no habitual exercise (coded as 4 dummy variables), logged real OOP costs during the past year.

Table 2 compares the prediction accuracies of the three RF models: full model (including all covariates), sub-model (1) (no difficulties with ADL), and sub-model (2) (difficulties with ADL or needing assistance). The lowest OOB error of the three models was 0.2229 for Model (1). This implies that the prediction accuracy of OOP costs for hypertensive patients without ADL difficulties was slightly better than that for all hypertensive patients who had continued their physician visits during the past two consecutive years.

**Table 2:** Comparison of prediction accuracy.

	All	No difficulties in ADL	Having difficulties in ADL or needing assistance
*N*	57,710	46,905	10,805
Features	45	25	35
OOB error	0.2355	0.2229	0.2956

**Figure 2:**
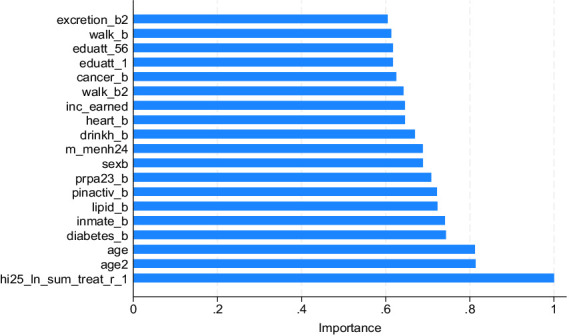
Variable importance of OOP costs (full model).

In the full model, the top 10 variables of importance were the highest quartile of OOP costs in the previous year, age, age squared, presence of diabetes, living together with family members excluding the spouse, having lipidemia, no habitual exercise, moderate or vigorous regular exercise, gender, moderate mental health status (Figure 2). Interestingly, most of these variables are consistent with the significant variables in the doubly robust estimation of Kumagai et al. (10), who analyzed the determinants of subjective poor health among hypertensive patients. This means that most determinants of poor subjective health and high medical costs are the same.

Hypertensive patients with diabetes or hyperlipidemia are often unaware of their symptoms and often allow their condition to progress/worsen untreated. As the above variables indicate, moderate or vigorous regular exercise is important to prevent the progression of arteriosclerosis, which is associated with poor subjective health. No habitual exercise is thought to contribute to the development of ischemic heart diseases such as angina pectoris and myocardial infarction.

**Figure 3:**
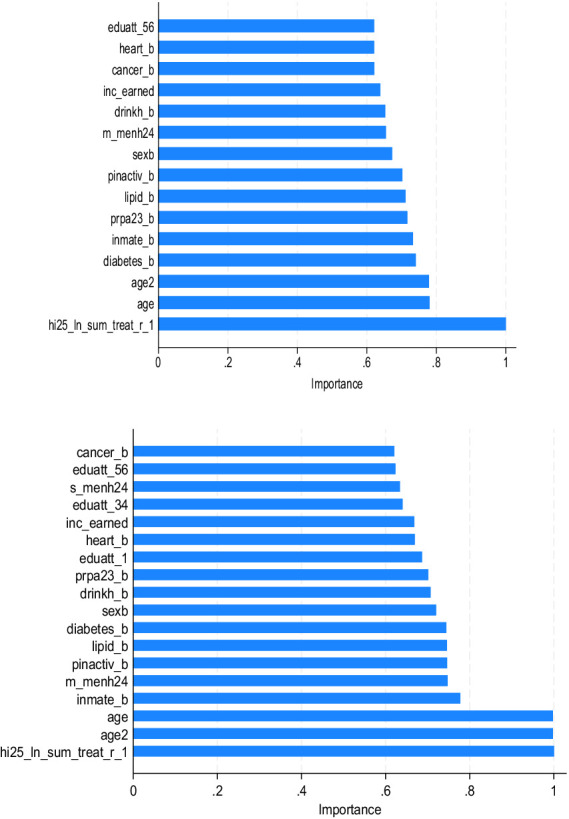
Variable importance of OOP costs [top: sub-model (1), bottom: sub-model (2)].

The sample was divided based on the presence or absence of difficulties in ADL. Sub-model (1) is the group with no ADL difficulty. The variables of importance for sub-models (1) and (2) are almost the same as those in the full model (Figure 3). However, in sub-model (1), the degree of importance of moderate or vigorous regular exercise is greater than that in the full model. This coincides with the results (28) showing that individuals with moderate or vigorous regular exercise tend to have a higher health stock. In contrast, the degree of importance of moderate mental health in sub-model (2) was greater than that in the full model. The lack of habitual exercise might have been associated with moderate mental health in the ADL difficulty group.

## 4 Discussion

Increasing the prevalence of complex diseases leads to more OOP payments (29). Furthermore, increases in OOP payments can have regressive effects, potentially leading to a substantial number of people being pushed into poverty due to health care costs (30, 31). Thus, accurate predictions of health spending patterns can help predict how countries will fare in the health care sector (31).

The determinants of OOP costs for lifestyle-related outpatients are not uniform, as some outpatients with hypertension also have other lifestyle-related diseases, such as diabetes (32). Indeed, according to the 2021 Survey on Trends in Lifestyle-Related Diseases (33), outpatients with diabetes or hypertension accounted for the first or second largest share of healthcare expenditures among 10 lifestyle-related diseases (34). The survey (33) shows that hypertension and hyperlipidemia had the first and second highest rates of physician visits, respectively, and the year-to-year increase in outpatient costs for diabetes and hyperlipidemia was above 5%.

Most contemporary health systems have achieved a certain degree of universal health coverage, particularly with regards to prevention, screening, diagnostics, and treatment of major NCDs such as hypertension (35). Given the long and clinically unpredictable course of the disease, most systems cover provision and reimbursement of essential medicines for hypertension and regular physician visits in primary care through the medical insurance premiums (36). However, sudden and predictable complications of hypertensive disease such as cerebral stroke, myocardial infarction or renal insufficiency, may result in life threatening conditions (37) with necessary hospital admissions to the intensive care unit (38). These treatments may last up to a few weeks and frequently lead to long-term impairment, disability, months-long absenteeism, decreased working ability and even premature mortality (39). Such consequences are the real-life toll and societal burdens of hypertension leading to huge OOP spending by patients and their families (40). Furthermore, this leads to the catastrophic household expenditure phenomenon which is widespread in the Global South LMICs countries without strong Japanese welfare and risk sharing agreements (41), which leads to families falling into debt and poverty traps (42). Therefore, we should carefully observe our Japanese OOP cost projections, particularly compared to Emerging BRICs Markets (43) as the leading global drivers of real GPD growth and consecutive health spending worldwide (44).

We estimated the predicted logged real OOP costs of hypertensive outpatients, with attention to complications of other lifestyle-related diseases and nonlinearities in the data, using RF models. The variables of importance shown in Figures 2, 3 indicate that preventing complications of diabetes or lipidemia is important for reducing OOP costs in outpatients with hypertension. Therefore, regular exercise of moderate or vigorous intensity is recommended in the no ADL difficulty group. In contrast, for hypertensive patients with ADL difficulties, habitual exercise must not be remedied to prevent the development of ischemic heart disease (45).

Providing accurate prediction of OOP payments for chronically ill patients and reducing the upper limit of OOP payments is important to prevent their catastrophic health care expenditures. However, due to data limitations, this study cannot link data on public pension benefits and OOP payments for older people to examine the desired upper limit of OOP payments.

Furthermore, it was well documented in the seminal literature that the importance of the education of patients about the risk factors may substantially influence their long-term behavior (46). Responsible attitude toward patient’s own disease may lead to precautious diet, exercise, healthy lifestyle and utilization of artificial intelligence assisted small, wearable medical devices (47). Such an equipment may provide the attending physician and the patient alike with reliable 24/7 supervision of the most, sensitive fluctuating clinical indicators such as arterial tension, cardiac rhythm, glycemia control or partial pressures of blood oxygen (48).

All these medical surveillance data are possible to be obtained to the scale of in-depth observation of inner bodily dynamics that was unthinkable only a few years ago. Constant education of health providers about the ongoing AI-related, technological revolution in clinical medicine is rapidly expanding the horizon of our understanding of arterial hypertension’s unpredictable dynamics in the real-world setting (49). Alongside this change, adoption of random forest and similar modeling approaches to the health econometrics field allow us to better cope with “a boomerang phenomenon.” Namely, rather insufficient density of physicians and nursing staff, have significant impact to the delayed discovery of hypertension persistence in the vast number of patients due to poor coverage of general population with screening measures (50).

In return this fact leads to a neglected, self-evolving disorder leading to microangiopathic changes of small blood vessels and accelerated atherosclerosis of large blood vessels (51). Both histopathological changes drive occurrence of unpredictable, severe and hard to treat clinical complications ranging from malignant cardiac arrhythmias, myocardial infarction, cerebral stroke etc. Most of these conditions require lengthy and exceptionally expensive intensive care unit admissions which might have been partially prevented with mass public health screening procedures of far more humble than overall budget impact (52). This is neglection of NCDs is widely known as the boomerang effect. Among other OECD countries the causal bottleneck insufficiency is prominent in Japan with 269 practicing physicians per 100,000 inhabitants as of 2020 (53). Room for successful intervention yet remains as witnessed by the findings of current study.

## 5 Conclusion

This study predicted the highest quartile group of OOP costs for hypertensive outpatients, focusing on complications with other lifestyle-related diseases and nonlinearities of the data. The estimation results of the RF models showed that the prediction accuracy of OOP costs for hypertensive patients without ADL difficulties was slightly better than that for all hypertensive patients who had continued their physician visits during the past two consecutive years. The important variables in the highest quartile of OOP costs were age, diabetes or lipidemia, lack of habitual exercise, and moderate or vigorous regular exercise. Habitual exercise must not be remedied to prevent the development of ischemic heart disease in patients with hypertension and ADL difficulties. In the present study, sub-model (2) recorded the highest OOB error of the three models; the mechanism by which the exclusion of older adults with no ADL difficulties contributed to the lower prediction accuracy is unclear. Future studies should be conducted to clarify this point.

## Data availability statement

The original contributions presented in the study are included in the article/Supplementary material, further inquiries can be directed to the corresponding author.

## Author contributions

NK: Writing – original draft, Formal analysis, Conceptualization. MJ: Writing – review & editing.
