# Supplementary material for: Random forest model used to predict the medical out-of-pocket costs of hypertensive patients
Source: Front Public Health. 2024 Jul 17;12:1382354. doi: 10.3389/fpubh.2024.1382354 (PMC11288809; doi:10.3389/fpubh.2024.1382354)
Supplement: Supplementary file 1 [file Table_1.docx]

**Table S1.** Non-response sample of copayments among hypertensive patients who continued physician visits during the past two consecutive years

|  | Variables | *N* | Mean or Proportion | SD | |
| --- | --- | --- | --- | --- | --- |
| Demographic variables | |  |  |  | |
|  | Age | 12,892 | 63.495 | 4.897 | |
|  | Dummy variable for aged 70-74 | 12,892 | 0.130 | 0.336 | |
|  | Gender (male=1) | 12,892 | 0.541 | 0.498 | |
|  | Married (reference) | 12,892 | 0.907 | 0.291 | |
|  | Never married | 12,892 | **0.093** | 0.290 | |
|  | Divorced or widowed | 12,892 | 0.000 | 0.022 | |
|  | Dummy variable for living together with family members excluding spouse | 12,875 | 0.547 | 0.498 | |
|  | Dummy variable for earned income during the past month | 10,658 | 0.640 | 0.480 | |
| Educational attainment | |  |  |  | |
|  | Junior high school | 12,892 | **0.219** | 0.413 | |
|  | High school (reference) | 12,892 | 0.497 | 0.500 | |
|  | Vocational school or junior college | 12,892 | 0.131 | 0.337 | |
|  | University or graduate school | 12,892 | 0.146 | 0.353 | |
| Sum of K6 | |  |  |  | |
|  | Serious mental health (12 < K6) | 8,499 | **0.080** | 0.271 | |
|  | Moderate mental health (4 < K6 ＜ 13) | 8,499 | 0.209 | 0.407 | |
| Objective health status | |  |  |  | |
|  | Dummy variable for having diabetes | 12,533 | 0.135 | 0.342 | |
|  | Dummy variable for having heart diseases | 12,485 | 0.080 | 0.272 | |
|  | Dummy variable for having lipidemia | 12,448 | 0.190 | 0.392 | |
|  | Dummy variable for having stroke | 12,380 | 0.047 | 0.211 | |
|  | Dummy variable for having cancer | 12,415 | 0.022 | 0.146 | |
| Perceived health | |  |  |  | |
|  | Dummy variable for felt worse symptoms of high blood pressure than its onset during the past year | 12,892 | 0.006 | 0.079 | |
| Lifestyle |  |  |  |  | |
|  | Drinking habit | 12,892 | 0.229 | 0.420 | |
|  | No habitual exercise | 12,794 | **0.390** | 0.488 | |
|  | Moderate or vigorous regular exercise | 12,892 | 0.267 | 0.442 | |
|  | Smoking habit | 12,892 | **0.193** | 0.395 | |
| Note: Non-respondents of OOP costs are excluded. | |  |  |  | |
| Sources: Longitudinal Survey of Middle-aged and Older Persons 2005–2020 | | | |  |  |
